# Mucosal Viruses in Myalgic Encephalomyelitis/Chronic Fatigue Syndrome: A Missing Piece of the Puzzle?

**DOI:** 10.3390/ijms262211161

**Published:** 2025-11-19

**Authors:** Krishani Dinali Perera, Paige Cameron, Tayyibah Sarwar, Simon R. Carding

**Affiliations:** 1 Food, Microbiome and Health Research Programme, Quadram Institute Bioscience, Norwich Research Park, Norwich NR4 7UQ, UK; krishani.perera@quadram.ac.uk (K.D.P.); paige.cameron02@icloud.com (P.C.); tayyibahh432@outlook.com (T.S.); 2 Norwich Medical School, University East Anglia, Norwich NR4 7UQ, UK

**Keywords:** myalgic encephalomyelitis/chronic fatigue syndrome (ME/CFS), viral persistence, DNA viruses, RNA viruses, mucosal reservoirs

## Abstract

Myalgic encephalomyelitis/chronic fatigue syndrome (ME/CFS) is a debilitating chronic condition without a definitive aetiology, no reliable diagnostic test, and no proven effective treatment. Despite most patients reporting a post-viral onset of illness, findings to date are conflicting on whether a single virus or multiple viral triggers are involved. Most studies to date have focused on detecting viruses in blood and circulating immune cells with relatively few investigating the presence of viruses in mucosal sites. In this review, we propose that this represents a critical gap in understanding the pathophysiology of ME/CFS knowledge, as mucosal tissues are primary entry points for most pathogens and often serve as reservoirs where viruses may persist. Consequently, they represent ideal niches for identifying persistent infections in ME/CFS. Emerging evidence from saliva and other mucosal samples in ME/CFS patients is consistent with this proposal and that latent viruses can persist and periodically reactivate in mucosal tissues from where they can potentially contribute to immune dysregulation, chronic inflammation, and increased symptom severity that defines ME/CFS.

## 1. Introduction

Myalgic encephalomyelitis/chronic fatigue syndrome (ME/CFS) is a debilitating condition affecting an estimated 70 million individuals worldwide [1]. It is characterised by persistent extreme fatigue, impaired sleep, dysautonomia, post-exertional malaise (PEM), and immune dysfunction. Overall, individuals with ME/CFS are more functionally impaired than those with other chronic conditions including multiple sclerosis, cancer, and rheumatoid arthritis [2,3]. ME/CFS can severely impact a person’s ability to work, attend school, participate in social activities, and perform daily tasks, with 75% of patients unable to work and 25% of individuals so severely affected that they become confined to their house or bed [3,4,5,6]. The absence of definitive biomarkers, reliance on self-reported symptoms, and heterogeneous pathologies further hinder progress in developing effective diagnostics and treatments.

A majority of patients report a post-infectious onset, often following a viral illness [7], with research suggesting that ME/CFS is a post-viral fatigue syndrome (PVFS) attributable to virus-driven dysregulation in immune function, neuroendocrine abnormalities, and impaired cellular energy metabolism [8,9,10,11]. Consistent with this hypothesis, persistent viral infections, including Epstein–Barr virus (EBV), human herpesvirus 6 (HHV-6), influenza, and enteroviruses, have been described in numerous studies of ME/CFS, although findings remain inconsistent. Establishing a viral origin and identifying consistent disease mechanisms are particularly challenging due to several obstacles: difficulties in comparing viral persistence between ME/CFS patients and healthy controls, the presence of latent viruses in asymptomatic individuals, underpowered cohort sizes, dependence on single samples or single time points which fails to recapitulate the natural progression of a virus infection or fluctuations and peaks of viraemia, and variability in testing methods [12,13]. Moreover, most of these studies have relied on blood samples, yielding inconsistent results.

Many blood-based virome studies to date report no significant differences in viral prevalence, viral load, or seroprevalence of viruses such as HHV-6, human herpesvirus 7 (HHV-7) or EBV between ME/CFS patients and healthy controls (Table 1). Importantly, the majority of high-throughput sequencing and broad virome analyses (Table 1) also show no distinct virus signature or increased abundance in ME/CFS blood samples. Although some studies report associations with viral reactivation or elevated antibody responses for herpesviruses and other viruses occasionally, these findings are not consistently replicated across cohorts. These variabilities can be attributed to differences in patient cohorts and diagnostic criteria (case definitions used), the sensitivity and specificity of detection methods and sample types (plasma, serum, PBMC), and the complexity of viral latency/reactivation states. Also, these studies overlook compartmentalisation and persistent mucosal viruses, which may obscure their contribution to ME/CFS disease mechanisms. Overall, the current literature underscores the heterogeneity of ME/CFS populations and the challenges and constraints of definitively linking viruses to ME/CFS aetiology based on blood-based virological markers alone [12].

Studies that have focused on mucosal viral persistence as a potential contributor to ME/CFS pathology are discussed in detail in Section 3. Mucosal viruses can persist within tissues such as the gastrointestinal (GI) and respiratory tracts, where they can evade immune surveillance and be undetectable in blood-based analyses. This hypothesis is further supported by comparisons with the PVFS, post-acute sequelae of SARS-CoV-2 infection (PASC), or long COVID, where clinical symptoms substantially overlap with ME/CFS diagnostic criteria, in which mucosal viral reservoirs have been described [34,35,36]. The aim of this review is to synthesise current evidence of mucosal viral persistence in ME/CFS, while discussing methodological approaches to study this important yet underexplored viral niche. Investigating mucosal viral reservoirs offers promising opportunities to clarify disease aetiology, establish biomarkers, and inform the development of targeted therapeutics, including re-purposing of licenced anti-viral drugs for ME/CFS.

## 2. Mucosal Immune System

The mucosal immune system maintains a delicate balance between pathogen surveillance and responsivity and immune tolerance to the complex communities of microbes (microbiomes) that cohabit mucosal sites across the body. It comprises a network of tissues collectively known as the mucosa-associated lymphoid tissue (MALT), which includes nasopharyngeal-associated lymphoid tissue (NALT), bronchus-associated lymphoid tissue (BALT), gut-associated lymphoid tissue (GALT), and other areas such as the conjunctiva-associated lymphoid tissue (CALT). The reproductive tract and genital mucosae, although lacking organised lymphoid follicles as in Peyer’s patches [37,38] contains specialised immune cells which in the female reproductive tract are regulated by sex hormones [39].

Within MALT, commensal microbes (the microbiota) and IgA play crucial roles in mucosal defence by restricting pathogen colonisation and neutralising them in the lumen, preventing their adherence to boundary epithelial cells, while limiting inflammatory responses [40,41]. The NALT serves as the first line of defence against airborne pathogens, with the connected BALT of the lower respiratory tract and lungs becoming activated during respiratory inflammation and infection. GALT, within the GI-tract (GIT), contains organised lymphoid structures such as Peyer’s patches, mesenteric lymph nodes, and isolated lymphoid follicles, and is one of the most well-studied and documented MALTs. Peyer’s patches are the primary site of mucosal immune responses within GALT, where B cells undergo activation and class switching, leading to the maturation of IgA-secreting plasma cells [42]. The intestinal epithelial barrier (IEB) provides an additional layer of protection and is composed of tight junctions, a mucus layer, and the lamina propria, which harbours diverse immune cell populations. Specialised epithelial cells further strengthen this barrier, where goblet cells secrete mucins that form a sterile mucus layer coating all epithelial surfaces, while Paneth cells produce antimicrobial peptides (AMPs), lysozymes, and defensins [43,44]. Innate lymphoid cells (ILCs), particularly ILC3, are abundant in mucosal tissues and produce cytokines such as IL-22 and IL-17 [45]. These cytokines promote epithelial cell differentiation, stimulate AMP secretion, and recruit neutrophils, thereby supporting epithelial repair and antimicrobial defence [45]. The lamina propria also contains adaptive immune cells, including different subtypes of CD4^+^ T helper cells (e.g., Th1, Th2, and Th17 cells, which secrete IL-17) and FoxP3^+^ regulatory T cells (Tregs), which maintain immune tolerance [46].

**Figure 1:**
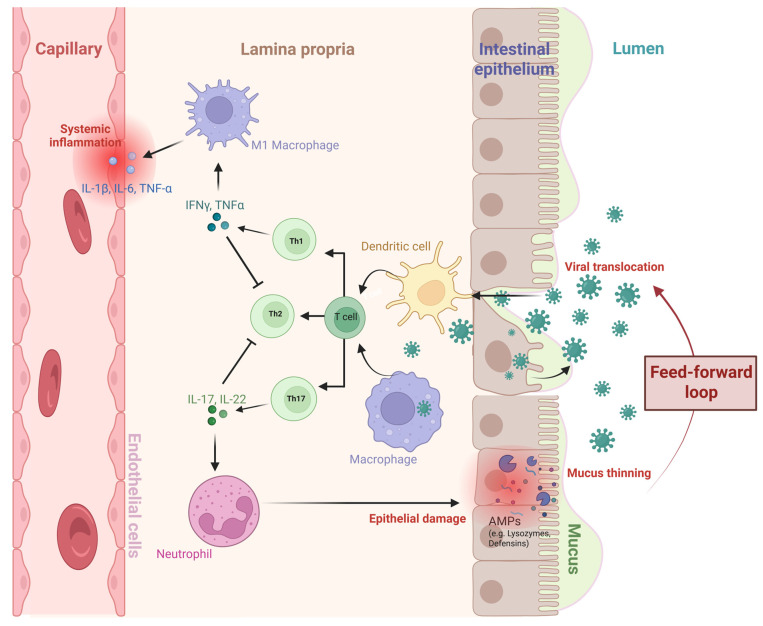
Conceptual schematic of the proposed role of persistent mucosal viruses in the aetiology of ME/CFS. Mucosal cells, such as epithelial cells, can serve as reservoirs for persistent viruses, potentially supporting viral replication under pathological conditions including stress, inflammation, microbiota dysbiosis, epithelial injury, or impaired immune surveillance [50]. These infected mucosal cells can release viral particles both apically (into the lumen) and basolaterally, promoting further infection and systemic spread [50,51,52]. Persistent mucosal viruses may also cross the disrupted gut intestinal epithelial barrier (leaky gut), activating dendritic cells and macrophages within the lamina propria, promoting CD4 Th1 and Th17 responses. Th1 cytokines, IFN-γ and TNF-α, activate M1 macrophages, driving the release of pro-inflammatory cytokines (IL-1β, IL-6, TNF-α) into systemic circulation. These cytokines inhibit Th2 differentiation or the production of immune tolerance promoting cytokines resulting in a more pro-inflammatory state dominated by Th1/Th17 responses, which further exacerbates inflammation. Th17-derived IL-17 and IL-22 promote neutrophil recruitment and the release of antimicrobial peptides (AMPs) such as lysozymes and defensins with chronic neutrophil activation leading to epithelial damage and degradation and thinning of the mucus layer. Disruption of the epithelial barrier facilitates viral translocation which may promote a feed-forward inflammatory loop perpetuating local and systemic immune dysregulation observed in ME/CFS. (Created in BioRender. Perera, K. (2025) https://BioRender.com/rxje1b4, accessed on 10 November 2025).

Mucosal barrier integrity can be compromised, particularly in the GIT, because of microbial dysbiosis, exposure to toxins, drugs or food/water-borne pathogens resulting in increased IEB permeability (“leaky gut”) and mucus thinning, reducing barrier functionality of the protective mucosal layer. This enables viral particles and microbial products to translocate across the epithelium and gain access to the systemic circulation, triggering immune activation, which, if sustained, leads to chronic inflammation (Figure 1) [47]. This pathway is relevant to ME/CFS, where IEB dysfunction, immune activation, and persistent low-grade inflammation are present and can contribute to disease pathophysiology [48]. Some viruses, such as EBV and HHV-6 [49], exploit mucosal immune architecture to establish long-term persistence within the host. Viral reactivation can provoke immune responses without achieving clearance, potentially sustaining the chronic immune activation frequently observed in ME/CFS patients.

## 3. Common Mucosal Viruses Associated with ME/CFS

Most ME/CFS patients recall an acute viral infection before disease onset [7,28], consistent with a viral aetiology. Historical outbreaks of infectious diseases have been followed by increased reports of ME/CFS-like symptoms, including polio virus, EBV, enteroviruses and influenza [53,54,55,56]. While eukaryotic viruses have been associated with ME/CFS or PVFS, most of these studies have relied on blood and analysis of peripheral immune cells. Several epidemiological and clinical observations (Table 2) suggest that mucosal viruses may contribute to disease onset and persistence.

### 3.1. Herpesviruses

Herpesviruses, such as EBV, HHV-6, and HHV-7, are double-stranded DNA viruses that can establish long-term latency following an acute infection. EBV causes infectious mononucleosis by infecting oropharyngeal epithelial cells and B lymphocytes and later establishes lifelong latency in memory B cells [65]. During asymptomatic reactivation, often triggered by psychological stress, EBV sheds in the oropharynx [66]. EBV-infected individuals show vulnerability to PVFS with a proportion fulfilling ME/CFS diagnostic criteria [67]. Of note, the vulnerability to post-EBV fatigue is more strongly associated with pre-existing symptoms and functional limitations than with initial immune or infection indicators [67], explaining perhaps why a subset of EBV-infected individuals might develop ME/CFS. Notably, elevated EBV-specific antibodies have been detected in the saliva samples of ME/CFS patients compared to controls [34], establishing the virus’s potential link to ME/CFS. In addition, sputum analysis of ME/CFS patients found that 85% released high viral loads of EBV, compared to 50% of age-matched healthy controls [59].

Other herpesviruses can persist in salivary glands and mucosal tissues, with HHV-6 DNA in ME/CFS patients being linked to mitochondrial dysfunction and altered immune responses [68]. HHV-7 shares tropism with EBV and HHV-6, though its role in ME/CFS remains less well studied. Importantly, findings regarding the association of mucosal HHV-6 with ME/CFS remain inconsistent. Herpesviruses including HHV-6, HHV-7 and EBV, along with papillomaviruses were more frequently detected in saliva from both ME/CFS patients and healthy controls with no significant difference in prevalence between the groups [31]. Another study reported no significant difference in HHV-6 or human cytomegalovirus (HCMV) viral loads between ME/CFS patients and healthy controls in sputum samples [59]. Similarly, a study investigating HHV-6 and HHV-7 in saliva samples found no difference in viral load between chronic fatigue syndrome (CFS) patients and healthy controls [21]. In contrast, a six-month longitudinal study of ME/CFS, found that those with severe symptoms exhibited more frequent and higher levels of HHV-6B and HHV-7 DNA in saliva compared to healthy controls [58]. In a subset of these patients, peaks in viral load coincided with symptom flare-ups across neurological, immune, autonomic, and other systems, suggesting that herpesvirus reactivation may contribute to disease pathology [58]. This finding indicates the importance of longitudinal sampling to accurately detect and capture the dynamics of viraemia, and suggests that in some ME/CFS patients, HHV-6B and HHV-7 may reactivate in mucosal tissues, potentially exacerbating symptoms. These observations are consistent with findings obtained from blood and plasma samples [18,27,29,33].

### 3.2. Parvoviruses

Parvovirus B19 may play a role in ME/CFS pathogenesis. A study of GI biopsies found that Parvovirus B19 DNA was markedly higher in CFS patients across all biopsy sites [61]. The same study also showed that HHV-7 was abundant, while EBV and HHV-6 levels were lower in these duodenal biopsies compared to the stomach biopsies in both CFS patients and controls [61]. Co-infections were similarly prevalent in both groups [61]. Interestingly, HHV-6 and Parvovirus B19 were not detected in PBMC samples from these parvovirus-positive patients, although EBV and HHV-7 were occasionally detectable in PBMCs of biopsy-positive individuals [61]. The inability to detect Parvovirus B19 in PBMCs indicates that the virus may persist primarily in mucosal tissues rather than circulating immune cells. This emphasises the importance of examining tissue-specific viral reservoirs in understanding ME/CFS viral pathogenesis.

### 3.3. Coronaviruses

The overlap between long COVID and ME/CFS highlights the importance of identifying mucosal viruses and their potential roles in ME/CFS disease mechanisms. SARS-CoV-2 infection can lead to long COVID in some patients which is estimated to affect at least 65 million individuals worldwide [69]; with the majority meeting the diagnostic criteria for ME/CFS and being more functionally impaired than long COVID patients without ME/CFS [70,71,72,73,74,75,76,77,78]. This suggests that PVFS develops in a subset of patients following SARS-CoV-2 infection, potentially linked to the reactivation of persistent viruses. Saliva antibody profiling revealed elevated responses to latent viruses, including EBV, HHV-6, and human endogenous retrovirus-K (HERV-K), in both non-vaccinated ME/CFS patients and healthy controls 3–6 months after mild or asymptomatic SARS-CoV-2 infection [34]. IgG levels against EBV antigens (EBNA1 and VCA) were significantly higher in ME/CFS patients, suggesting enhanced viral reactivation [34]. These findings indicate that even mild infections may trigger latent virus reactivation, contributing to immune dysregulation in ME/CFS. Additionally, evidence of persistent or reactivated mucosal viruses months after SARS-CoV-2 infection supports the hypothesis that latent virus reactivation at mucosal sites may contribute to ME/CFS onset. Importantly, these elevated antibody levels were more prominent in saliva and not detectable systemically in plasma, highlighting the importance of assessing local mucosal immunity [34]. Similarly, most COVID patients with persistent long COVID symptoms, including fatigue, cognitive impairment, headaches, and sleep disturbances, showed evidence of EBV or HHV-6 reactivation, as indicated by the detection of viral DNA in saliva and the oropharynx [36]. Among this study cohort, patients with herpesvirus reactivation (EBV, HHV-6 or coinfection of EBV and HHV-6) experienced a more severe form of COVID-19, including higher rates of pneumonia and longer hospital stays, compared to patients without viral reactivation [36]. These findings emphasise that reactivation of herpesviruses may exacerbate the severity of long COVID symptoms in patients. Also of note, a Norwegian study found that individuals infected during the 2009 H1N1 influenza pandemic had more than a two-fold increased risk of developing ME/CFS compared to non-infected individuals, although laboratory confirmation of active influenza infection was limited [54]. Thus, respiratory viruses of epidemic potential could drive disease pathologies like those observed in ME/CFS. Further longitudinal studies are essential to track the disease course and determine if affected patients will eventually develop ME/CFS.

### 3.4. Picornaviruses

Enteroviruses are members of the *Picornaviridae* family, which are single-stranded RNA viruses with GI and respiratory tropisms. These viruses can persist in deep tissues, such as skeletal muscle and the brain, potentially provoking chronic low-grade inflammation and immune dysregulation [79]. Historical epidemics of ME/CFS have shown acute symptoms coinciding with enterovirus outbreaks [79,80,81,82]. Enterovirus RNA has been detected in stomach biopsies from CFS patients [60], as well as in throat swabs [57]. In one report, three patients with documented acute enterovirus infection subsequently developed ME/CFS where stomach biopsies taken years later revealed persistent, low-abundance enteroviral RNA and capsid protein [62]. This indicates the potential for enteroviruses to persist long-term in mucosal epithelial cells following acute infection and provides evidence that it may contribute to the pathophysiology of ME/CFS.

### 3.5. Adenoviruses

Adenoviruses are double-stranded DNA viruses that infect respiratory and GI epithelia. While acute adenovirus infections are often self-limiting, evidence suggests that adenoviruses can persist in the NALT, remaining latent for months to years [83]. Adenoviruses in the context of ME/CFS, however, remain understudied. Notably, COVID-19 infection may trigger adenovirus reactivation in the oral mucosa of ME/CFS patients, as indicated by elevated IgG levels against Human adenovirus (HAdV) detected in saliva following SARS-CoV-2 infection [35]. This IgG elevation was not detected in plasma [35]. Interestingly, in another study by the same group, HAdV was detected in sputum from a patient with severe ME/CFS and in a non-ME/CFS immunosuppressed individual, but not in other ME/CFS patients or healthy controls [59]. These results, while intriguing, are insufficient in determining whether HAdV contributes to the pathophysiology of ME/CFS, particularly in severe cases.

### 3.6. Retroviruses

Another important family of viruses that may contribute to immune dysfunction in ME/CFS are HERVs, such as HERV-K, which can be detected in a variety of tissues, including mucosal tissues [84,85]. HERVs are retroelements derived from ancient infections of germ cells by exogenous retroviruses during evolution and now comprise nearly 8% of the human genome (“fossil viruses”). They can contribute to the pathophysiology of human disease, with their expression linked to a wide range of conditions, including autoimmune diseases such as multiple sclerosis [86,87,88,89,90], cancers such as melanoma and prostate cancer [91,92,93,94], and inflammatory bowel disease [95]. HERV expression is tightly regulated but influenced by host antiviral immunity and exogenous viral infections, which can trigger (re)activation [96,97]. Relevant viruses that trigger (re)activation of HERV expression include SARS-CoV-2 [98,99,100,101,102], HHV-6 [88], EBV [89,103], and HIV [104], the consequences of which include impaired physical function, inflammation, disease severity, and subsequently immune senescence [105,106]. To date, findings regarding HERVs in ME/CFS are inconsistent. In one study, HERV-K transcript levels in PBMCs did not differ between healthy controls and ME/CFS patients, and no correlation was observed between HERV-K18 transcript levels and HHV-6 or HHV-7 viral copy number [21]. By contrast, differential expression of HERV-K, but not HERV-W, has been reported in the blood of patients with moderate ME/CFS [107]. Likewise, a genome-wide HERV expression profiling of PBMCs revealed distinct, disease-specific HERV signatures that distinguished ME/CFS patients from healthy controls and fibromyalgia patients with overlapping symptomology and chronic fatigue with ME/CFS, with the most pronounced HERV dysregulation correlating with symptom severity [108]. Overexpression of HERVs of the H, K, and W types has also been detected in a small cohort of fibromyalgia patients, with or without comorbid ME/CFS, with increased HERV expression levels correlating with interferon-β and interferon-γ expression levels [109]. These findings indicate a potentially disease-relevant dysregulation of HERVs in ME/CFS. Yet, evidence from mucosal samples remains sparse. One study revealed the presence of different HERVs (HERV-K, HERV-FRD and HERV-R) in duodenal biopsies from 8 out of 12 ME/CFS patients [63]. Supporting this evidence, saliva antibody profiling revealed elevated responses to HERV-K, along with other latent viruses including EBV and HHV-6, in both non-SARS-CoV-2 vaccinated ME/CFS patients and healthy controls months after mild or asymptomatic SARS-CoV-2 infection [34]. Given that mucosal areas are primary sites of viral persistence and immune activation, investigating HERV expression and antibody responses to HERVs in these compartments could provide new insight into the role of HERVs in ME/CFS.

### 3.7. Gastrointestinal Tract Viruses

Our own study of the GI (faecal) virome in ME/CFS and healthy individuals found that while the virome and bacterial diversity were similar between ME/CFS patients and controls, certain human viruses, including HHV-6A, papillomaviruses, coronavirus NL63, and adenovirus 54, were detected exclusively in ME/CFS samples [64]. This suggests subtle but distinct viral–bacterial host associations specific to ME/CFS that needs to be confirmed in larger studies.

### 3.8. Reproductive Tract Viruses

It is regrettable that the reproductive tract has received limited attention in ME/CFS research. Persistent viral infections in reproductive tissues have the potential to affect fertility, alter hormone production, increase the risk of viral transmission both vertically and sexually, and provoke local inflammation and immune activation. Numerous viruses are known to persistently infect and establish reservoirs in male and female reproductive tissues, including CMV, HHV-6, mumps virus, herpes simplex viruses (HSV-1 and HSV-2), human T-lymphotropic virus (HTLV), human papillomavirus (HPV), hepatitis B and C viruses, as well Zika virus and Ebola virus [110,111,112]. The effects of SARS-CoV-2 on reproduction are still unclear. Notably, the immune-privileged nature of the male reproductive tract could facilitate viral persistence. Thus, it is possible that viruses capable of persisting in the reproductive tract are not only sexually transmissible but may also play a role in the development of ME/CFS. However, there is currently no evidence linking viral reservoirs in the reproductive tract to ME/CFS, highlighting the need for further investigation.

Given that ME/CFS is more prevalent in females [7,113,114], it is important to investigate the sex-specific differences in the reproductive tract that may contribute to disease susceptibility or progression. The female reproductive tract (FRT) is a microbially diverse environment, and the mucosal surfaces contain a spectrum of antimicrobial factors that serve as the first line of defence against invading pathogenic bacteria, viruses, and fungi [115]. The antimicrobial defences throughout the FRT are regulated in part by sex hormones, which influence their production by innate immune cells [115]. Most research on the FRT has focused on the bacteriome, while relatively few studies have investigated the viral components within this ecosystem. The FRT harbours abundant bacteriophages which constitute the majority of the prokaryotic virome, and this reflects the structure of the bacterial community which may influence disease-associated shifts in the microbiome [116,117]. A diverse range of eukaryotic viruses are also present in the FRT with *Papillomaviridae*, *Anelloviridae*, and *Orthoherpesviridae* viruses being the most prevalent [116]. DNA eukaryotic viruses in the FRT are linked to adverse outcomes such as preterm birth, inflammation, and cervical cancer [116] while little is known about RNA viruses. This again highlights the need for further research into the role of the FRT virome in health and disease, and particularly its possible contribution to the higher prevalence of ME/CFS in females.

Collectively, the findings from virus studies in ME/CFS underscores that virus persistence in mucosal sites is often ignored or understudied. Post-viral triggers such as SARS-CoV-2, and evidence of their persistence and reactivation support the assumption that mucosal viral reservoirs contribute to ME/CFS pathophysiology. Subtle differences in viral composition, as observed in the GI virome, and detection of specific DNA viruses exclusively in ME/CFS patients further suggest that viral–host interactions at mucosal sites could influence and be a driver of immune dysregulation, chronic inflammation, and symptom severity. The systematic investigation of mucosal viruses as potential drivers or modulators of ME/CFS is warranted for further study.

## 4. Mucosal Viral Persistence in ME/CFS: A Hypothesis

The evidence from ME/CFS, long COVID and other PVFS points towards a shared aetiology: that certain viruses remain active or reactivate in mucosal tissues, influencing both local and systemic immune responses. In ME/CFS sites such as the oral cavity, nasopharynx, gut, or reproductive tract can serve as long-term viral reservoirs. Similarly, persistence of replicating virus, or their nucleic acid/antigens are linked to long COVID [118]. As highlighted in Section 3, multiple viruses have been identified to persist in mucosal sites, with increases in viral load correlating with symptom flare-ups [58]. Notably, increased replication of persistent viruses following SARS-CoV-2 infection [34,35,36] was detected exclusively in saliva but not plasma samples [34], and patients with persistent viral reactivation exhibited more severe COVID [36]. These findings suggest that mucosal compartments may harbour clinically relevant viral activity often missed by blood-based studies, underscoring the need for more focused research on these mucosal reservoirs.

**Figure 2:**
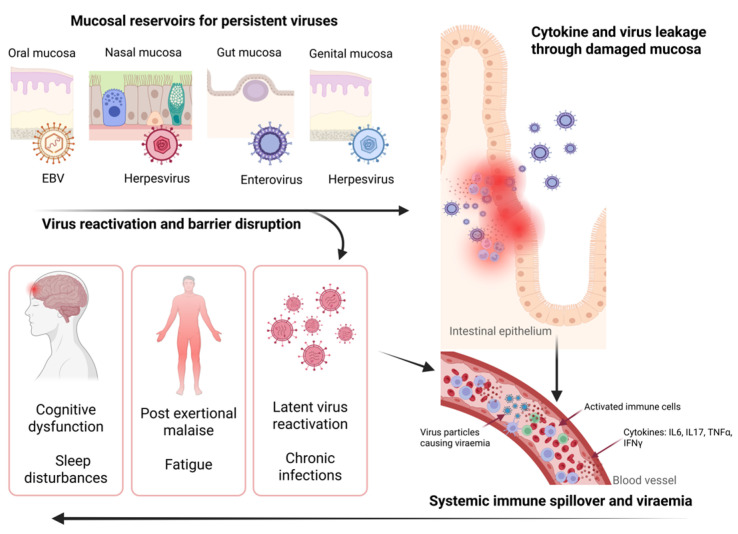
A proposed mechanism linking latent mucosal viral reservoirs to systemic inflammation and neuroimmune symptoms observed in ME/CFS. Latent viruses such as EBV, HHV6/7, and enteroviruses persist in mucosal tissues. Reactivation triggered by internal or environmental stressors, combined with immune-mediated damage to the epithelial barrier, disrupts its integrity. Immune spillover occurs as local pro-inflammatory mediators (e.g., cytokines) and immune responses escape the mucosal compartment, leading to the release of cytokines and viral leakage into deeper tissues and bloodstream causing viraemia. The resulting systemic inflammation promotes chronic low-grade immune activation. Concurrent viral infection in endothelial cells (e.g., herpesviruses) may induce endothelial dysfunction and cause vascular abnormalities, further exacerbating systemic inflammation. Circulating neuroimmune factors could reach the central nervous system, causing neuroinflammation. Disruption of the hypothalamic–pituitary–adrenal (HPA) axis impairs cortisol production and stress regulation, often because of ongoing inflammation. Persistent viral activity and inflammation can also activate HERVs further amplifying immune dysregulation and contributing to chronic infection. All these factors drive key ME/CFS symptoms, including fatigue, cognitive dysfunction (brain fog), post-exertional malaise, and sleep disturbances. This model highlights how mucosal reservoirs may contribute to systemic immune dysregulation in ME/CFS. (Created in BioRender. Carding, S. (2025) https://BioRender.com/5bmmgg5, accessed on 10 November 2025).

Viruses such as EBV and HHV-6 can remain active in mucosal or tissue reservoirs without being detectable in the circulation at the time of sampling. Since many of these virus suspects are common in healthy, asymptomatic individuals, detecting their presence in blood at a single time point does not accurately reflect their pathogenic potential. Overall, persistent viral activity in these sites can maintain the local immune system in a state of low-level activation, eventually disrupting normal regulation and contributing to immune exhaustion, fatigue, cognitive deficits, and post-exertional malaise. We have termed this the “mucosal viral persistence in ME/CFS” hypothesis (Figure 2). This hypothesis can explain why blood-based studies often yield inconsistent results.

Persistent virus infection in mucosal tissue drives chronic immune activation, producing pro-inflammatory cytokines and immune mediators. Chronic inflammation could disrupt tight junction proteins, and increased epithelial barrier permeability (“leaky barriers”) would allow viral particles, microbe-derived microvesicles, particulate antigens, and inflammatory mediators to access the systemic circulation, thereby exacerbating the immune response. Impaired mucosal immune response, microbiome dysbiosis, and persistent virus triggered inflammation can impact the autonomic nervous system through neuroendocrine pathways. Herpesviruses such as EBV and HHV-6 can also infect endothelial cells leading to endothelial dysfunction characterised by microvascular abnormalities and microclot formation, which may underlie cognitive impairments and neurological disturbances observed in ME/CFS [119]. Moreover, latent herpesvirus antigens could inhibit apoptosis of these infected endothelial cells, prolonging dysfunction and contributing to hypercoagulability, vascular pathology, and compromised vascular integrity [119]. Although mucosal virus reactivation might be sufficient to induce neuroinflammation, some neurotropic herpesviruses may directly reactivate within the central nervous system, further driving neuroinflammation and contributing to chronic relapse-recovery cycles by facilitating infection across a dysfunctional blood–brain barrier [118,120]. Additionally, persistent virus infections could disrupt the hypothalamic–pituitary–adrenal (HPA) axis, leading to abnormal cortisol production and stress response [121,122] where the severity of the disruption could be influenced by pre-existing HPA abnormalities or the magnitude of the response to the virus infection [122]. Oxidative stress induced by chronic inflammation may further impair mitochondrial function, contributing to overall fatigue and other hallmark symptoms of ME/CFS such as cognitive dysfunction, and post-exertional malaise.

It is important to acknowledge the absence of direct experimental or clinical evidence that demonstrates the causal mechanisms of mucosal virus persistence in ME/CFS. This significant knowledge gap underscores the need for future investigations involving mucosal sampling and multi-omics approaches to understand virus persistence, immune interactions and ME/CFS pathogenesis.

## 5. Methodological Directions

A multi-disciplinary approach is essential to investigate viruses within mucosal sites in well-defined clinically diagnosed cohorts of patients, and appropriately matched healthy and disease control cohorts and to validate the theory of mucosal virus persistence in ME/CFS. Importantly, non-invasive sample collection at mucosal sites offers the practical advantage of remote, home-based, sampling that enables severely affected ME/CFS patients to contribute to research studies. Furthermore, swab samples may accurately reflect viral communities in relevant tissues affected by ME/CFS pathophysiology, replicating viral presence and potential reactivation in the mucosal reservoirs that blood-based studies miss. In addition, repeated sampling to capture virus activity over time is feasible with such non-invasive, home-based sampling protocols, acknowledging that it can increase cost and participant burden. It is worth noting the limitations of mucosal swabs and, in particular, their typically low viral particle/nucleic acid yield, which can increase the risk of false negatives and require highly sensitive, carefully optimised, uniform and controlled methods for extraction and detection in addition to inclusion of appropriate control samples to distinguish genuine signals [123].

Quantitative PCR approaches are well-established methods for quantifying viral loads with high sensitivity for known viral sequences. Additionally, non-biassed metagenomic-based next-generation sequencing enables a comprehensive study of viral communities within samples, including de novo detection of viruses. Although comparatively and subjectively less sensitive than qPCR, metagenomic next-generation sequencing is valuable in characterising a multitude of viruses in complex biological samples, including the broader virome and uncovering co-infections or rare viral species. However, sample contamination is a particular concern for low biomass samples and highly sensitive sequencing methods require strict laboratory controls to exclude, for example, “kitomes” from downstream analyses. Importantly, detecting the presence of RNA viruses in virome analysis is challenging owing to RNA instability and limitations in sequencing approaches and data interpretation [124]; thus, their role is often underestimated.

By combining targeted detection methods with broader sequencing approaches and aligning sampling with disease onset and symptoms, a more comprehensive picture of how mucosal viral activity relates to ME/CFS can be established. This integrated approach would also help distinguish between transient exposure, latent infection, and active replication of mucosal viruses which may be associated with the pathogenesis of ME/CFS.

## 6. Conclusions

While recent advances have highlighted the role of viral infections, particularly EBV, HHV-6/7, influenza, and SARS-CoV-2, in the onset and chronicity of ME/CFS, much of the research has to date focused on blood-based investigations and single-time point analyses, leaving the role of the mucosal immune system largely unexplored. Emerging evidence from saliva, mucosal tissues, and longitudinal studies suggests that latent viruses can persist and periodically reactivate at mucosal sites, potentially driving immune dysregulation, chronic inflammation, and symptom severity via mechanisms involving gut–brain axis interactions and local immune responses. These findings, along with observations in long COVID and other PVFS, emphasises that viral “reservoirs” at mucosal surfaces may have lasting impacts beyond initial infection.

To address current knowledge gaps, future research should consider repeated mucosal sampling, combined virus detection methods including metagenomic sequencing, and comprehensive immune and symptom tracking to clarify whether mucosal viral activity is a causative agent, a consequence, or a bystander in ME/CFS. Confirming the clinical relevance of mucosal viral persistence could enable the use of non-invasive sampling methods, the discovery of robust biomarkers, and the development of targeted antiviral and immunomodulatory therapies, as well as drug repurposing (e.g., anti-retroviral therapy; ART) clinical trials. This could ultimately improve diagnosis and symptom management for individuals suffering from this complex and debilitating disease.

## Figures and Tables

**Table 1:** Summary of blood-based virus studies in ME/CFS.

Sample	Diagnostic Criteria	Cohorts	Detection Method	Viruses Studied *	Association with ME/CFS	Ref.
Serum	Centers for Disease Control and Prevention (CDC)	548 chronic fatigue syndrome (CFS) patients and 30 healthy controls	Immunoassay	Herpes simplex virus (HSV) 1/2 Rubella Adenovirus HHV-6 EBV Cytomegalovirus (CMV) Coxsackie B virus types 1–6	No difference in the seroprevalence of the viruses between CFS and healthy controls.	[14]
Serum	Oxford	8 CFS patients	PCR and sequencing	Enterovirus	Enteroviruses can persist in the serum of CFS patients.	[15]
Serum and peripheral blood leukocytes (PBLs)	CDC	26 CFS patients and 50 healthy controls	PCR and antibody profiling	HHV-6A/B HHV-7	No difference in active or latent HHV-6A/B or HHV-7 infections between CFS patients and controls.	[16]
Serum, plasma and PBMC	CDC-1994 (“Fukuda”)	35 CFS patients (27 severe CFS) and 25 healthy controls	PCR and immunoassay	HHV-6 HHV-7 HHV-8	Frequent reactivation of HHV-6 detected in CFS patients.	[17]
PBLs and plasma	Fukuda	17 diagnosed CFS patients, 12 unexplained CFS patients, and 20 healthy controls	PCR and immunoassay	HHV-6 HHV-7	No difference in prevalence of HHV-6 or HHV-7 between the groups. CFS patients showed a significantly higher rate of dual HHV-6 and HHV-7 infection, increased plasma viraemia for HHV-7 and simultaneous reactivation of both viruses.	[18]
PBLs and plasma	Fukuda	108 ME/CFS patients and 90 healthy controls	PCR and immunoassay	HHV-6 HHV-7 Parvovirus B19	Active HHV-6, HHV-7 and parvovirus B19 infections and co-infection of these viruses show a high prevalence in ME/CFS patients compared to healthy controls.	[19]
Serum	Fukuda	72 CFS patients and 59 healthy controls	Immunoassay	HHV-6A/B	No difference in antibody levels to HHV-6A/B between CFS patients and healthy controls.	[20]
PBMC	Fukuda	39 CFS patients and 9 healthy controls	RT-PCR	HHV-6 HHV-7 Human endogenous retrovirus (HERV)-K18	No difference in HHV-6 or HHV-7 viral copy numbers or HERV-K18 transcripts between CFS patients and healthy controls.	[21]
Plasma	Canadian Consensus Criteria (CCC)	25 ME/CFS patients, and 25 healthy controls	Metagenomics	Broad virus diversity	No consistent viral associations detected in ME/CFS and contamination and batch effects confound results if controls are inadequate.	[22]
Whole blood	CCC	25 ME/CFS patients, and 25 healthy controls	Metagenomics	Anelloviruses Pegivirus 1 Herpesviruses Papillomaviruses	No difference in the viral composition between ME/CFS cases and controls.	[23]
Serum	CCC	163 ME/CFS patients with/without comorbidities and 103 healthy controls	Immunoassay	HHV-1 to HHV-7	No significant difference in IgG reactivity to HHV-1 to HHV-7 between ME/CFS and controls, but there are subtle variations in antibody responses to HHV-1 and EBV antigens in ME/CFS.	[24]
Whole blood	CCC	14 female ME/CFS patients and 11 matched sedentary controls	Metagenomics	Enterovirus A Influenza A Anelloviruses Human herpesviruses	No differences in viral abundance or virome composition between ME/CFS patients with post-exertional malaise and controls before or after exercise.	[25]
Plasma	CCC and Fukuda	251 ME/CFS patients and 107 healthy controls	Immunoassay	HSV-1/2 VZV CMV EBV HHV-6	No difference in the seroprevalence of these viruses between ME/CFS patients and healthy controls.	[26]
PBMC and plasma	Fukuda	58 ME/CFS patients and 50 healthy controls	PCR and immunoassay	HHV-6 CMV EBV	Active EBV infection is more common in ME/CFS patients, with no significant differences in active CMV or HHV-6 infections.	[27]
Plasma	Fukuda or CCC	226 ME/CFS patients and 99 healthy controls	Immunoassay	HSV-1/2 VZV CMV EBV HHV-6	Herpesvirus serology could potentially distinguish different subgroups of ME/CFS patients based on their reported disease trigger (infection versus non-infection)	[28]
PBMC and serum	Fukuda	30 ME/CFS patients and 20 healthy controls	PCR and immunoassays	HHV-6 HHV-7 EBV	Higher frequencies of both latent and active HHV-6, HHV-7, EBV, and co-infections of these viruses detected in ME/CFS patients compared to healthy controls.	[29]
Whole blood	Fukuda	134 ME/CFS patients and 33 healthy controls	PCR	HHV-6A/B	Disease severity in ME/CFS relates to persistent HHV-6 viral load.	[30]
Plasma and PBMC	Fukuda and CCC	391 ME/CFS patients and 292 healthy controls	PCR, and VirCapSeq	Herpesviruses Pegiviruses Anelloviruses HERVs	No difference in the prevalence or diversity of viruses between ME/CFS patients and healthy controls.	[31]
Plasma	CDC or CCC	222 ME/CFS patients and 46 multiple sclerosis (MS) patients	Immunoassay	HSV-1/2 VZV CMV EBV HHV-6	IgG antibody concentrations explained symptom associations in multiple sclerosis more robustly than in ME/CFS.	[32]
Whole blood and plasma	Fukuda	200 ME/CFS patients and 150 healthy controls	PCR	HHV-6A/B HHV-7 Parvovirus B19	Persistent active infections or co-infections with HHV-6A/B, HHV-7, and parvovirus B19 are more frequent in ME/CFS patients compared to healthy individuals.	[33]

* Refers to all viruses investigated in each study, including detection of viral antigens, antibodies, nucleic acids, or other viral markers as applicable. Specific viral components assessed (e.g., antigen vs. antibody) may vary by study and are detailed in the cited publications.

**Table 2:** Publications on persistent viral analysis in mucosal regions of ME/CFS patients.

Mucosal Sampling Site	Diagnosis Criteria	Cohorts	Detection Method	Viruses Studied *	Association with ME/CFS	Ref.
Throat swab	Oxford	175 throat swabs from CFS patients	PCR and sequencing	Enteroviruses	Enterovirus sequences found in some CFS patients.	[57]
Saliva	Fukuda	39 CFS patients and 9 healthy controls	RT-PCR	HHV-6 HHV-7	No difference in HHV6 or HHV7 viral loads. No difference detected in human endogenous retrovirus-K18 (HERV-K18) levels in PBMCs.	[21]
Saliva	CCC and/or Fukuda	14 ME/CFS-Mild/ Moderate, 16 ME/CFS-Severe Patients, and 16 healthy controls	ddPCR	HHV-6B HHV-7 HSV-1 EBV	High viral loads of HHV-6B and HHV-7 observed in ME/CFS patients, and fluctuations in viral load correlated with specific ME/CFS disease phenotypes. No difference in EBV or HSV-1 viral loads.	[58]
Saliva	CCC	95 ME/CFS patients (78 female) and 110 healthy controls (71 female)	Antibody profiling	EBV HHV-6 HERV-K	COVID-19 triggers reactivation of latent herpesviruses (EBV, HHV-6) and HERV-K, with stronger reactivation and uniquely elevated EBV nuclear antigen antibodies in ME/CFS patients, who also show higher baseline EBV antibodies before SARS-CoV-2 infection.	[34]
Saliva and oropharynx		88 post-COVID patients (68 with herpesvirus reactivation and 20 non-detectable herpesvirus DNA controls). 46/88 female and 42/88 male.	RT-PCR	EBV HHV-6	Herpesvirus reactivation is associated with post-COVID syndrome and chronic fatigue.	[36]
Saliva	CCC	84 ME/CFS patients and 94 healthy controls	Antibody profiling	Adenovirus SARS-CoV-2	SARS-CoV-2 triggers Adenovirus reactivation in ME/CFS.	[35]
Saliva and faeces	Fukuda and CCC	106 ME/CFS patients and 91 healthy controls	VirCapSeq	Herpesviruses and papillomaviruses	No difference in the prevalence of viral sequences between ME/CFS patients and healthy controls.	[31]
Sputum	CCC	13 ME/CFS patients, 10 healthy, 4 elderly, and 2 immunosuppressed controls	RT-PCR	EBV HHV-6 Adenovirus	High EBV viral load in ME/CFS patients. No difference in HHV-6 viral loads.	[59]
Stomach biopsies	CDC	165 patients, 22 healthy controls, and 12 patients with other gastric disorders	Immuno-staining	Enteroviruses	Enterovirus detected in stomach biopsies from 82% of CFS patients compared to 20% of controls.	[60]
Gastric antrum and duodenum biopsies	Fukuda	48 CFS patients and 35 healthy controls. 78% patients and 66% of the controls were females.	RT-PCR	HHV-7 HHV-6 EBV Parvovirus B19	High viral loads of Parvovirus B19 detected in ME/CFS patients.	[61]
Stomach biopsies		Follow-up of three patients with acute enterovirus infection, later developed ME/CFS	RT-PCR Immuno-staining	Enteroviruses	Acute enterovirus infection could lead to ME/CFS, and enteroviruses were detected in stomach biopsies years after initial infection.	[62]
Duodenal and stomach biopsies	CCC and Fukuda	12 ME patients and 8 healthy controls	Immuno-staining	HERVs	Immunoreactivity to HERV proteins was observed in some ME/CFS patients.	[63]
Faeces	CCC and National Institute for Health and Care Excellence (NICE) 2007/CG53 guideline	9 severely affected female ME/CFS patients and 8 healthy controls (3 males and 5 females)	Virome profiling, metagenomics	Multiple bacteriophages HHV-6A Papillomaviruses Coronavirus NL63 Adenovirus 54	ME/CFS-specific viral–bacterial associations.	[64]

* Refers to all viruses investigated in each study, including detection of viral antigens, antibodies, nucleic acids, or other viral markers as applicable. Specific viral components assessed (e.g., antigen vs. antibody) may vary by study and are detailed in the cited publications.

## Data Availability

No new data were created or analyzed in this study. Data sharing is not applicable to this article.
